# Effect of a Superabsorbent Polymer (Poly-Gamma-Glutamic Acid) on Water and Salt Transport in Saline Soils under the Influence of Multiple Factors

**DOI:** 10.3390/polym14194056

**Published:** 2022-09-27

**Authors:** Yuliang Fu, Shunsheng Wang, Shikai Gao, Songlin Wang, Zhikai Gao, Zhenjia He

**Affiliations:** 1 School of Water Conservancy, North China University of Water Resources and Hydropower, Zhengzhou 450045, China; 2 Shaanxi Provincial Land Engineering Construction Group Co., Ltd., Xi’an 710075, China

**Keywords:** HYDRUS-1D, poly-γ-glutamic acid, soil water infiltration, solute (Cl^−^) transport, sandy loam soil

## Abstract

In order to effectively suppress the negative effects of salt ions contained in saline soils on agricultural soil quality and crop growth, this study took advantage of the water-saving properties and better soil improvement properties of poly-γ-glutamic acid (γ-PGA). By carrying out various experiments, the following relationships have been found. (1) The lab experiment studies the effect of the γ-PGA application on the infiltration of sandy loam soil. The application rates of γ-PGA are 0%, 0.1%, 0.2%, and 0.3%, respectively. (2) HYDRUS-1D is used to simulate water infiltration of sandy loam soil under multiple factors (bulk density, γ-PGA application rate, and the application depth of γ-PGA). (3) The effect of γ-PGA on soil solute (Cl^−^) transport is also explored in this paper. The results show that bulk density and the application depth of γ-PGA (*p* < 0.01) have higher effects on cumulative infiltration than the application amount of γ-PGA (*p* < 0.05). A lower γ-PGA application rate will increase the proportion of unavailable soil water by 3%. The established empirical models have good results. Furthermore, when the γ-PGA application rate is 0.3% (0.02-cm^2^ min^−1^), the Cl^−^ hydrodynamic dispersion coefficient is the highest. The study recommends applying the γ-PGA at 1.4 g cm^−3^, 5–20 cm, and 0.2%. The results of this study are conducive to an in-depth understanding of the physicochemical properties of poly-γ-glutamic acid, improving the utilization rate of salinized land, achieving agricultural water and fertilizer conservation and yield enhancement, and guaranteeing sustainable land use and sustainable development of agroecological environment.

## 1. Introduction

Salinization is one of the typical factors of soil degradation in arid and semi-arid regions worldwide [1]. It severely threatens global agricultural production and food security [2]. In recent years, due to the combination of unique climatic and soil characteristics and anthropogenic activities, the water resources of the Yellow River in China are becoming increasingly scarce [3,4]. The Yellow River water has become an available but unreliable source of moisture; coupled with the fact that Henan Province in China is a province with high grain production capacity, the vast majority of its irrigated agricultural areas are located in the Yellow River basin, with soft geology [5], poor soil water retention capacity [6], unreasonable use of chemical fertilizers and insufficient groundwater supply, which can easily lead to soil salinization and secondary salinization [7], mainly in the following ways. (1) The former saline soils contain salt in the whole layer; the most apparent feature is the salt surface aggregation, white saline patches visible in the topsoil nowadays the salt is pressed below the crop root layer and becomes dark alkali [8]; (2) the distribution of saline soils has changed from whole to local. In fact, as a result of saline land improvement, soil salinity is only controlled below the crop root layer, or the micro-environment of the plant root distribution layer is improved. Traditional salt washing and salt pressing improvement measures only drench the soil salinity down to the soil layer that can be reached by irrigation, under which there is always a large amount of salt, and it is difficult to move the salt. In saline soil areas to promote water-saving irrigation technology, there are apparent salt accumulation and desalination areas in the soil body [9]; (3) the agricultural hazard from “extinction” ring “yield reduction” change. With the improvement of irrigation technology and agricultural farming, the harm of soil salinization to agricultural production has changed from the initial lack of harvest to the current reduction of crop yield, directly threatening national food security, land quality, and sustainable use [10]. In response to the harmful effects of salt content in saline soils on soil quality and crop growth, physical, chemical, biological, and agricultural engineering remediation measures suitable for improving saline soils have been sought. Among the various methods and standards, applying soil conditioners is one of the effective measures to restore saline soils and rapidly improve the soil environment. Polyglutamic acid (γ-PGA) is a natural polymer made from L-glutamic acid and D-glutamic acid through dehydration and condensation, produced by the fermentation of Bacillus subtilis ZJS18. It has the ability to inhibit soil water evaporation, improve soil water storage capacity, slow down the rate of water release and prevent soil water infiltration and loss. It also has a solid ability to balance acidity and alkalinity and can effectively balance the soil PH value to avoid acidification and caking of agricultural soil caused by the long-term use of chemical fertilizers. In evaporative infiltration comparison experiments found that only single adding different mass ratios of γ-PGA SAP significantly prolonged the change of soil water from FC to PWP and reduced the infiltration of standing water in sandy loam soils, thus reducing the deep seepage losses of soil water. Therefore, it is considered that SAP can be used in large quantities in the field of agricultural production in sandy loam soils [11]. The results found that the heavy metal Cu^2+^ ions in the wastewater could be effectively removed by poly-γ-glutamic acid (γ-PGA), and better adsorption could be obtained at an initial solution pH = 5.5. The static adsorption isotherm behaviour of γ-PGA was consistent with the Langmuir model, which had a good fit, and further exploration seems to be needed in the following aspects. In addition to this, poly (gamma-glutamic acid) (γ-PGA) is considered to be a promising fertilizer synergist in agricultural systems and plays an important role in the soil nitrogen (N) cycle [12]. A potted study using 15 N-labeled CO(NH_2_)_2_ and (NH4)_2_SO_4_ to investigate the fate of these two fertilizers in soil planted with Brassica chinensis with and without addition of γ-PGA, and the results found that γ-PGA addition reduced the overall loss of CO(NH_2_)_2_-N and (NH)_2_SO_4_-N by 6.93% and 3.54% of applied N, respectively, and reduced the soil N loss by 2.65% and 1.63% of applied CO(NH_2_)_2_-N and (NH)_2_SO_4_-N, respectively. Several studies have demonstrated that the N cycling of CO(NH_2_)_2_ was changed by the addition of γ-PGA [13,14,15]. Zhang [15] found that γ-PGA addition reduced soil NH_4_^+^-N content which was conducive to delayed formation of NO_3_^−^-N, and this would be helpful to temporarily store more available N in soil for plant uptake. To better understand the role of γ-PGA in promoting plant growth, used the effects of labelled γ-PGA synthesized from 13C1-15N-Lglutamicacid (L-Glu) on soil nutrient availability, plant nutrient uptake capacity, plant metabolism and its distribution in the plant-soil system. The study results found that γ-PGA significantly increased plant yield and N, P, K nutrient uptake by strengthening the uptake capacity of roots and regulating the nutrient availability through changing microbial and enzymatic characteristics [16]. γ-PGA can immobilize Cd in soil and form dissolved complexes with heavy metals to clean heavy metal-contaminated soils of Cu, Zn, Ni and Cr [17,18]. r-PGA can also stabilize Cd and Pb in the soil and weaken their bioavailability, thereby reducing their uptake and accumulation by crops [19]. As heavy metals are not easily removed or destroyed by natural mechanisms, they persist in the subsurface environment for a long time after their release [19,20]. In the remediation of heavy metal-contaminated soils, γ-PGA applications can be used to reduce the risk to human health and ecosystems [21,22]. Researchers have shown through the halophytes Sesbania and Sedum that they have moderate salt tolerance, high productivity and economic and ornamental value [23,24]. A pot experiment was conducted to investigate whether the application of γ-PGA could improve the removal of Ca^2+^, Mg^2+^ and NO_3_ by halophytes. After 56 days of application of 1000 mg L^−1^ γ-PGA, 93.25%, 94.78% and 84.26% were achieved for Ca^2+^, Mg^2+^ and NO_3,_ respectively. In addition, application of γ-PGA alleviated salt stress, promoted plant growth and improved antioxidant performance. Plant growth and improved antioxidant performance.

We have noticed that there are some studies on the application of γ-PGA to control heavy metal pollution in agricultural fields and salt tolerance in saline crops, but there are few studies on the effects of γ-PGA on infiltration characteristics and solute migration in saline fields, and the results were only analyzed for soils with single-factor physical property changes, neglecting the effects of γ-PGA on infiltration characteristics and solute migration under the interaction of various soil physical factors (soil bulk density, depth of γ-PGA application, γ PGA content), the influence of γ-PGA on the infiltration characteristics and solute migration patterns. Once the bulk density of the soil, the depth of application of γ-PGA and the content of γ-PGA change simultaneously, γ-PGA will inevitably affect the changes in soil texture, soil structure, solute type, pore water velocity and soil porosity, and the combination of these factors will have a new effect on the infiltration characteristics of the soil and the saline soil [25]. The combination of these factors will have a novel effect on soil infiltration characteristics and the effect of salinity in saline soils [26]. Therefore, the aim of this study was to investigate the effect of γ-PGA on the infiltration characteristics and salt transport in saline soils and the changes in its parameters under the interaction of multiple soil parameters. Among these, the soil parameters of saline soils include the depth of application of bulk density γ-PGA, the variation of γ-PGA content.

This paper combines soil column infiltration with numerical simulation (Philip model and HYDRUS-1D model) to study the effects of the hydrogel made by γ-PGA, including water absorption property, infiltration capacity and Cl^−^ transport. The aim is to find a reasonable method of γ-PGA utilization. The above results can provide a theoretical basis for soil improvement techniques.

## 2. Materials and Methods

### 2.1. Experimental Materials

#### 2.1.1. Soil Sample

The test soil was obtained from the sandy loam of the demonstration base of micro-irrigated date palm in Yuanzhi Mountain, Mizhi County, northern Shaanxi, China. Due to the occurrence of different degrees of secondary soil salinization, 30 square areas of about 2 m^2^ were randomly selected in the field due to typical salinized farmland, about 10 cm of soil was removed from the cultivated layer, leveled with a small shovel, and 20 cm deep soil samples were taken. The soil samples were dried in a cool place in the laboratory, air-dried, and the dead branches and remaining materials were removed from the soil samples and sieved through 2 mm sieve. Soil volumetric water content was determined by the EC-5 soil moisture monitoring system (METER Inc., Pullman, WA, USA); subsequently, soil samples were ground and extracted at a soil-water mass ratio of 1:5, and the conductivity of the solution was determined using a DDS-307 conductivity meter (INESA Inc., Shanghai, China) and converted to obtain the soil salinity [27,28], with two replications for each treatment. Soil pH values were measured using pH paper (LMAI Bio Inc., Shanghai, China).

**Figure 1:**
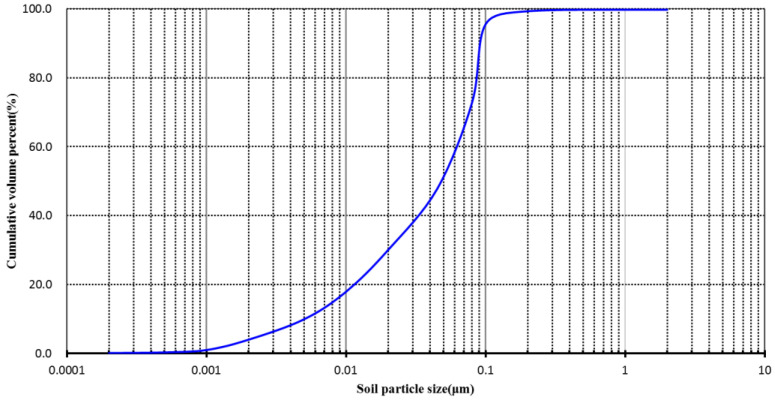
Grain size distribution curve of the soil.

Results of soil particle gradation composition are shown in Figure 1 (the international system was used to classify the soil texture). Basic composition and physical properties characteristics of soils for the test are shown in Table 1.

After analysis, the percentage of soil grains by volume were 69.5% in sand, 29.0% in silt and 1.5% in clay, which classified this soil as sandy loam according to the analysis of soil texture by the international system.

#### 2.1.2. Physical and Chemical Properties of γ-PGA

**Figure 2:**
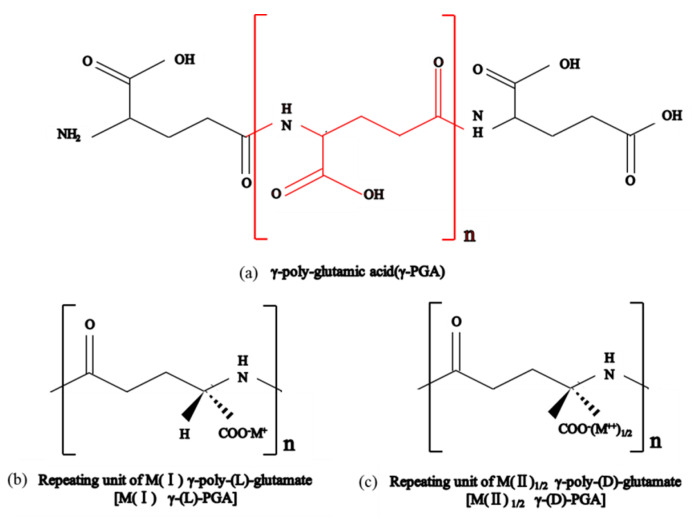
Molecular structure formula of γ-PGA.

Poly-glutamic acid (γ-PGA) is a polypeptide molecule composed of D-glutamic acid (D-Glu) and L-glutamic acid (L-Glu) monomers linked by an amide bond between a-amino and y-carboxyl groups. The molecular structure formula of γ-PGA is shown in Figure 2. γ-PGA is a water-soluble, biodegradable, non-toxic polymer. The γ-PGA is provided from FREDA biotechnology Co., Ltd. (Ji’nan, China). γ-PGA is nontoxic white powder with a molecular weight of 700 kD and can be degraded for two years in soil. In the indoor test, 2 g of γ-PGA is placed in the dialysis bag (m_1_), and the mass of the additive and dialysis bag is m_2_, and the bag is sealed at both ends and put into a beaker filled with 1000 mL of distilled water, and then the bag is taken out of the beaker and placed in suspension for 15 min after 12 h. After there are no more water drops, the mass is m_3_, and the water absorption multiplier is calculated as (m_3_–m_2_)/(m_2_–m_1_), and the test is repeated three times in each group, and the average value of the water absorption multiplier of γ-PGA in distilled water is 731 g/g, and that in tap water is 319 g/g [29].

### 2.2. Experimental Method and Measurement

#### 2.2.1. The Measurement of Soil-Water Retention Curves (SWRC)

The soil-water retention curves influenced by γ-PGA are measured by high-speed soil centrifuge using H-1400PF (Kokuan Chemical Co., Ltd., Chuo-ku, Tokyo, Japan).The results are fitted to the equations of retention curves by VG model [30,31]. The constitutive relationship by the RETC code is developed by van Genuchten et al. [32].

(1) $$S_{e}=\frac{\theta -\theta_{r}}{\theta_{s}-\theta_{r}}=\;{\left(1+|\alpha h{|}^{n}\right)}^{-m}\;\;\;\;\;\;\;\;\;\;\;\;\;\;\;\;\;\;\;\;\;h>0$$

(2) $$\;\;\;\;\;\;\;\;\;\;\;\;\;\;\;\;\;\;\;\;\;\;\;\;\theta =\theta_{s}\;\;\;\;\;\;\;\;\;\;\;\;\;\;\;\;\;\;\;\;\;\;\;\;\;\;\;\;\;\;\;\;\;\;\;\;\;\;\;\;h\;\leq \;0$$

(3) $$\begin{array}{c} \;\;\;\;\;\;\;\;\;\;\;\;\;\;\;\;\;\;\;\;\;\;\;\;K\left(h\right)=\frac{K_{s}{\left\{1-{\left(\alpha h\right)}^{nm}{\left[1+{\left(\alpha h\right)}^{n}\right]}^{-m}\right\}}^{2}}{{\left[1+{\left(\alpha h\right)}^{n}\right]}^{ml}} \end{array}$$

In equation from (1) to (3), where $S_{e}$ is effective saturation; *h* is the soil-water pressure head (cm); θ is the volumetric water content (cm^3^·cm^−^^3^); $\theta_{r}$ and $\theta_{s}$ are the residual and saturated moisture content (cm^3^·cm^−^^3^), respectively; α, m, n and l are empirical parameters and m=1−1/n; l is found to be equal to 0.5 for most soils; and $K_{s}$ is the saturated hydraulic conductivity (cm·min^−^^1^).

#### 2.2.2. Soil Infiltration Experiments under Single Factor Influences

The experiments are contrast experiments with four treatments with the first experiment to be replicated three times. γ-PGA powders with different application rates (0% (CK, which is the control group), 0.1%, 0.2%, and 0.3%) are mixed evenly with soil samples. Soil samples are added into a transparent acrylic column (100 cm length, 18 cm inner diameter) for 90 cm in height with three soil densities (1.3 g cm^−^^3^, 1.35 g cm^−^^3^ and 1.4 g cm^−^^3^). The water supply device is a Mariotte bottle (5 cm inner diameter, 50 cm height). The pressure head is 2.5 cm. The measure probes of soil water (METER Inc., Pullman, WA, USA) are located at 10 cm, 20 cm, 30 cm, 40 cm, 50 cm, and 60 cm below the soil surface. The infiltration continues for 360 min. The cumulative infiltration, wetting front and soil moisture content are the leading observation indicators in this experiment. The experiments include three factors (bulk density, γ-PGA application rate, and the application depth of γ-PGA) and three levels of orthogonal test (Table 2). No. 1–No. 9 are simulated by HYDRUS-1D model. No. 10–No. 12 are completed in the laboratory.

#### 2.2.3. Soil Solute (Calcium Chloride) Transport Experiments

**Figure 3:**
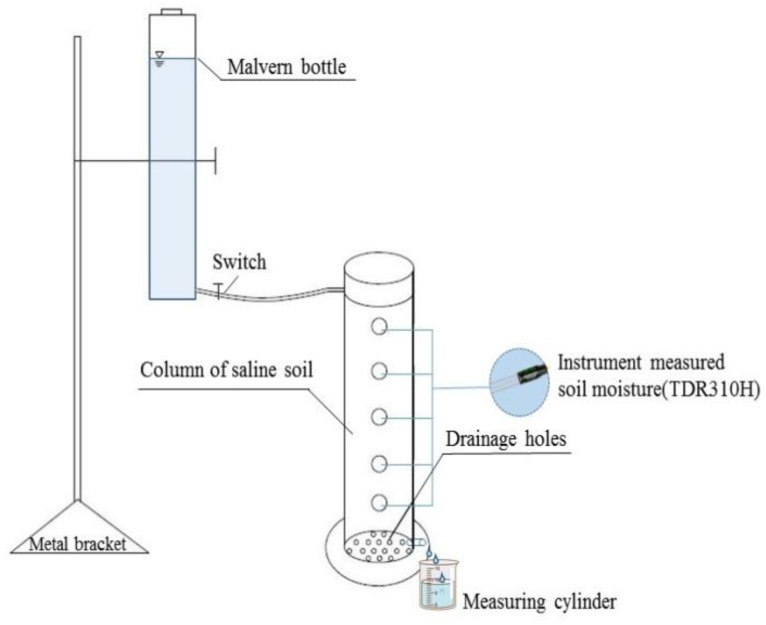
Device for infiltration and its composition.

**Figure 4:**
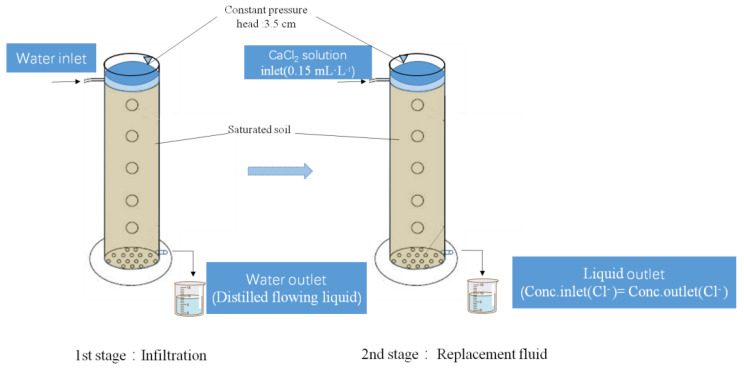
Diagram of the process of using the device.

The γ-PGA application rates are 0% (CK), 0.1%, 0.2%, and 0.3% (mass ratio). The bulk density is 1.35 g cm^−^^3^. The transparent acrylic column is 40 cm long and its inner diameter is 5 cm, with a constant pressure head of 3.5 cm. The infiltration of distilled water is completed when the soil reaches saturation. Then, the replacement fluid (0.15 mL L^−^^1^ CaCl_2_ solution) is used for infiltration. A measuring cylinder is put at the bottom of the soil column to receive the flowing liquid. The infiltration of 0.15 mL L^−^^1^ CaCl_2_ solution is completed when the Cl^−^ concentration of the flowing liquid is equal to that of the replacement fluid. The schematic diagrams regarding device for infiltration and its composition is shown in Figure 3 and the schematic diagrams regarding diagram of the process of using the device is shown in Figure 4.

### 2.3. Evaluation Criteria

To evaluate the model performance, the root mean square error (*RMSE*), the sum of squares due to error (*SSE*), the mean absolute error (*MAE*) and the Mean Error (*ME*) are used [33]:

(4) $$RMSE=\sqrt{\frac{1}{N}\overset{N}{\underset{i=1}{\sum }}{\left(y_{i}-{\hat{y}}_{i}\right)}^{2}}$$

(5) $$SEE=\frac{1}{N}\overset{N}{\underset{i=1}{\sum }}{\left(y_{i}-{\hat{y}}_{i}\right)}^{2}\;$$

(6) $$MAE=\frac{1}{N}\overset{N}{\underset{i=1}{\sum }}{\left|y_{i}-{\hat{y}}_{i}\right|}^{}\;$$

(7) $$ME=\frac{1}{N}\overset{N}{\underset{i=1}{\sum }}(y_{i}-{\hat{y}}_{i})\;$$

In equation from (4) to (7), where *N* is sample size, and$\;y_{i}$ and ${\hat{y}}_{i}\;$are real and predicted values, respectively. In addition, the coefficient of determination (also called *R*^2^) was used to evaluate the model performance:

(8) $$R^{2}=\frac{\sum_{i=1}^{N}(y_{i}-{\hat{y}}_{i})}{\sum_{i=1}^{N}{\left(y_{i}-\bar{y}\right)}^{2}}\;$$

In equation (8), where $\bar{y}$ is the mean value of the true data. The coefficient of determination shows how much of the variability in true values can be caused by the relationship to the predicted values. *R*^2^ is represented by a value up to 1: *R*^2^ = 1 means the model predicts data with a perfect fit, with 0 the model always predicts the mean value, and negative values mean the model cannot predict the data.

The Pearson’s method is used for correlation calculations. Pearson’s correlation coefficient is defined as [34]:

(9) $$r_{XY}=\frac{\sum_{i=1}^{N}\left(X_{i}-\bar{X}\right)\left(y_{i}-\bar{Y}\right)}{\sum_{i=1}^{N}{\left(X_{i}-\bar{X}\right)}^{2}\sum_{i=1}^{N}{\left(y_{i}-\bar{Y}\right)}^{2}}\;$$

In Equation (9), where $X_{i}$ and $Y_{i}$ are the individual sample points, and $\bar{X}$ and $\bar{Y}$ are the sample means. Pearson’s correlation coefficient $r_{XY}\;$ranges from −1 to 1, where −1 and 1 indicate perfectly negative and positive linear correlations, respectively, and 0 indicates nonlinear correlation.

To compare Pearson’s correlation coefficients with the variable importance measures (which range from 0 to 1), the correlation coefficients are normalized by dividing the coefficients by their sum and taking the absolute value. The specific relevant formula is shown in Equation (10):

(10) $$r_{}\;{}_{XY_{i}}^{norm}=\frac{\left|r_{XY_{i}}\right|}{\sum_{i=1}^{N}r_{XY_{i}}}\;$$

### 2.4. Soil Infiltration Model

The relationship between cumulative infiltration and time is described by Philip infiltration model as follows [35]:

(11) $$I=St^{0.5}+At^{}$$

In Equation (11), where I is cumulative infiltration (cm); S is sorptivity (cm·min^−^^0.5^); and A is steady infiltration rate (cm·min^−^^1^).

#### HYDRUS-1D Model [36]:

HYDRUS-1D is adopted to simulate 1D infiltration under the influence of multiple factors (bulk density, γ-PGA application rate and the application depth of γ-PGA). The HYDRUS-1D code is based on 1D Richards’s equation under homogeneous and isotropic soil, which can be described as:

(12) $$\frac{\partial \theta \left(h,t\right)}{\partial t}=\frac{\partial }{\partial z}\left[K\left(h\right)\left(\frac{\partial h}{\theta z}+1\right)\right]$$

**Figure 5:**
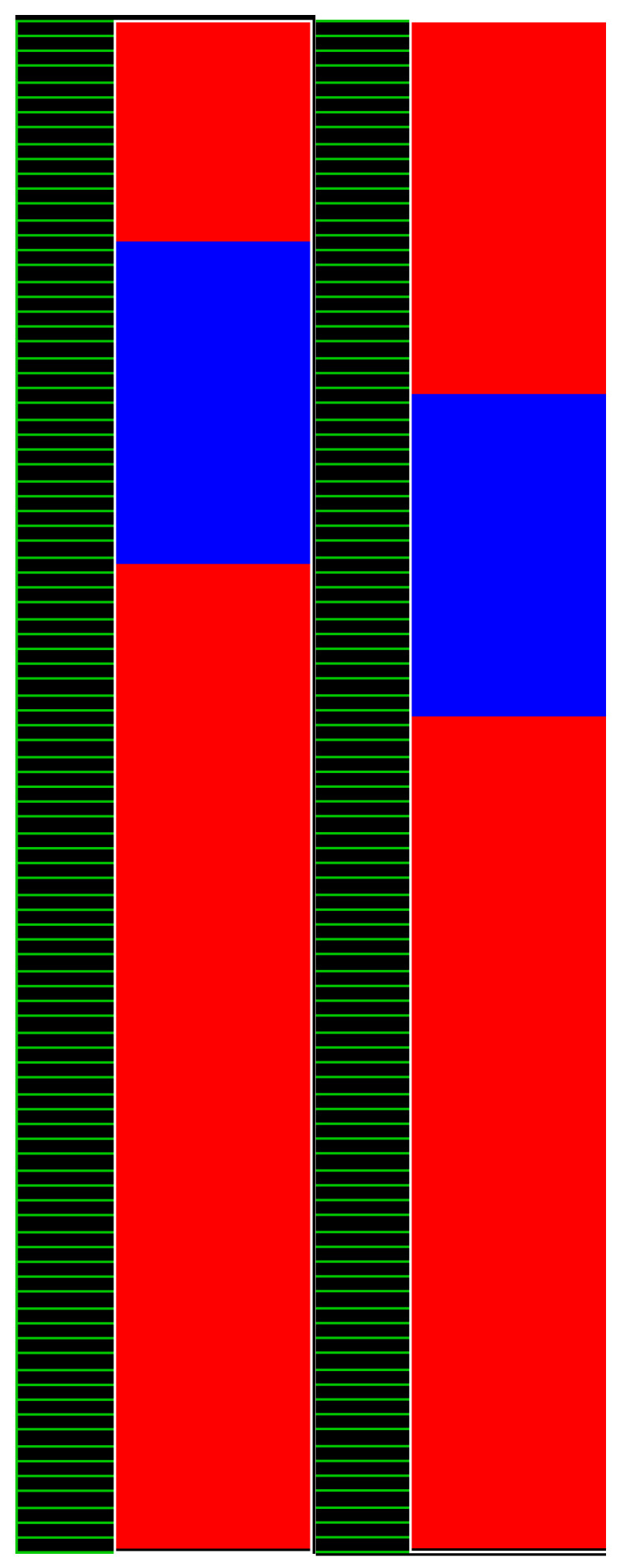
The initial and boundary conditions.

In Equation (12), where *t* is time (min); *z* represents the vertical coordinate with the origin at the soil surface (positive upward) (cm), and the *K(h)* denotes the unsaturated hydraulic conductivity (cm·min^−^^1^) determined by Equation (3). Figure 5 shows the initial and boundary conditions in this study.

The initial conditions are as follows:

(13) $$\theta =\theta_{0},h=-2.5\mathrm{cm},0\leq z\leq 100\mathrm{cm},t=0$$

In Equation (13), where $\theta_{0}$ is the initial soil moisture before irrigation (cm^3^·cm^−3^).

The boundary conditions are as follows:

(14) $$\{\begin{array}{c} h=-2.5,z=0,t\geq 0\\ \frac{\partial h}{\partial z}=0,z=100,t\geq 0 \end{array}$$

### 2.5. Statistical Analysis

The data are processed by MS Excel, Origin 8.0, MATLAB, and IBM SPSS 25.0. Multiple comparisons are performed with the LSD method. The equations of retention curves of the γ-PGA-treated soil are fitted by RETC code. Both the retention curves and the measured amount of cumulative infiltration are entered into the HYDRUS-1D model. The inversed solution in HYDRUS-1D and the Levenberg–Marquardt optimization method are used to optimize the soil hydraulic parameters. The corresponding soil hydraulic parameters of the soil mixed with different γ-PGA content are presented in Table 3. The objective function is defined as the sum of squared residuals (*SSQ*). The correspondence between simulated and observed data are evaluated by correlation coefficient (*R^2^*) at *p* = 0.05, and the root mean square error (*RMSE*).

## 3. Results and Discussion

### 3.1. Effects of γ-PGA on Soil Infiltration Characteristics under a Single Factor

#### 3.1.1. Cumulative Infiltration and Infiltration Rate

**Figure 6:**
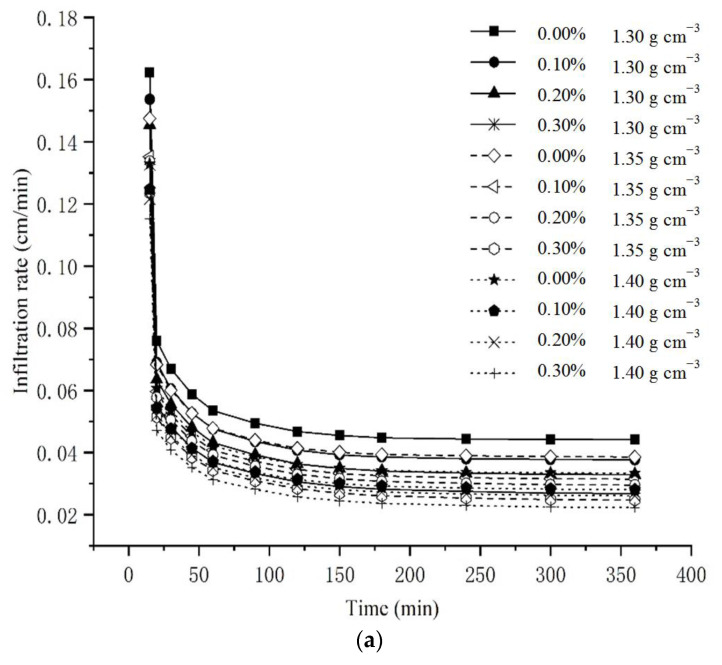

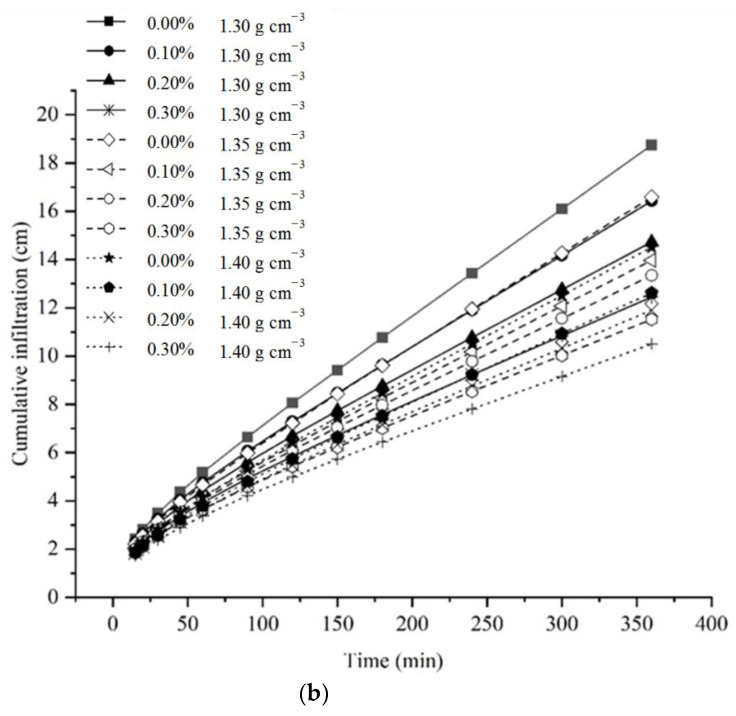
The infiltration capacity under single factor. (**a**) The cumulative infiltration under single-factor. (**b**) The infiltration rate under single-factor.

According to Figure 6a,b, there are few differences among the treatments at the beginning of infiltration (15–30 min). This is due to the extremely high absolute value of soil matric potential (SMP) of air-dried soil (unsaturated). Moreover, as an effect of intermolecular forces, water in contact with the solid phase wets it and covers the solid phase with a thin layer of water. As time goes by, the water potential gradient sharply declines and so does the infiltration rate. This is when the differences become apparent. A cumulative infiltration of 90 min can be used as an indicator to reveal the initial soil infiltration capacity [37]. Compared with CK, the cumulative infiltration of 1.3 g cm^−3^ and 0.3% γ-PGA treatment, the cumulative infiltration of 1.35 g cm^−3^ and 0.3% γ-PGA treatment, and the cumulative infiltration of 1.4 g cm^−3^ and 0.3% γ-PGA treatment at 90 min have decreased by 25.9%, 23.5% and 20.8%, and the final cumulative infiltration dropped by 33.6%, 30.6% and 27.7%, respectively. The application of 0.3% γ-PGA significantly reduces the infiltration. 

#### 3.1.2. Infiltration Parameters

According to Table 4, the infiltration characteristics of γ-PGA-treated soil can be well-described by the model. The stable infiltration rate (*A*) and the infiltration and adsorption rate (*S*) decline with the increase of γ-PGA application rate and bulk density. Lower S indicates less absorption capacity of γ-PGA-treated soil, with better ability to temporarily store water. The quantitative relationship between the infiltration parameters of Philip model and γ-PGA application rate is assessed to analyze the influence of γ-PGA on soil infiltration (Table 5). There is a good exponential relationship among these parameters (*R*^2^ higher than 0.86).

According to Table 3, saturation moisture content (SMC) decreases with the rising application amount of γ-PGA. Additionally, n is soil dehydration rate which increases when bulk density increases and the application amount of γ-PGA decreases. This is because γ-PGA-treated soil will swell after absorbing water, resulting in larger soil pores and lower bulk density [19].

**Figure 7:**
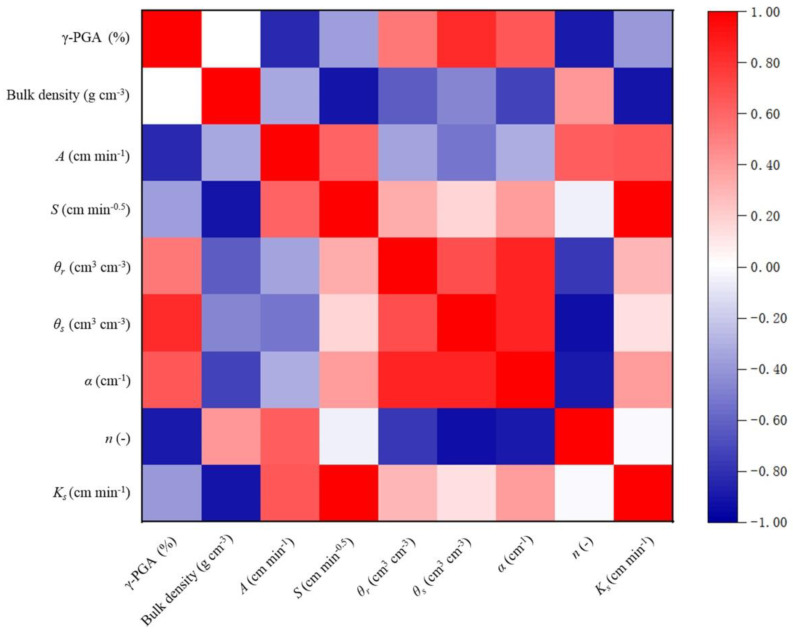
Heatmap of correlations in the acquired dataset.

For additional analysis, the correlation coefficients, representing the relationship between two variables, were obtained using Pearson’s method. The heatmap of correlation coefficients for the mechanical properties is shown in Figure 7.

Figure 7 shows the interrelationship between hydraulic parameters using HYDRUS-1D model to invert the γ-PGA-treated soil and Philip model parameters with γ-PGA content. γ-PGA content is positively correlated with a and *θs* (*p* < 0.5), and negatively correlated with steady infiltration rate A and n (*p* < 0.5), whereas soil capacitance is significantly negatively correlated with parameters *θr*, *α*, *Ks*, and *S* (*p* < 0.5). The increase in γ-PGA content causes the soil to absorb a large amount of water, which increases the water-holding capacity of soil particles and increases the saturation water content. In addition, the significant positive correlation between γ-PGA content and the shape parameter A of the soil moisture characteristic curve indicates that the inlet suction value of saturated soil decreases, at which time, due to the low value of inlet suction, when the soil water changes from saturated to unsaturated stage, it is difficult to drain water from the soil and the soil always maintains a high water content. The significant negative correlation between γ-PGA content and steady infiltration rate A of the Philip infiltration model indicates that under the unit potential gradient, the flow through unit soil area decreases, the hydraulic conductivity decreases, and the infiltration volume is small, especially water in the infiltration process; because γ-PGA content improves the soil structure, the water flow through the small pore aperture decreases, and the infiltration path is forced to change direction. γ-PGA effectively improves the soil structure; the soil particles continuously fill the small pore size, the water flow through the small pore size decreases, forcing the infiltration water to change direction, the infiltration flow path increases and the flow velocity is slow and tortuous, which has an obvious inhibiting effect on the deep seepage characteristics of soil water, which is biased towards the lighter soil with smaller bulk weight, and the more obvious the influence of γ-PGA has on the soil water-holding capacity [38,39].

#### 3.1.3. Soil Water Holding Capacity

**Figure 8:**
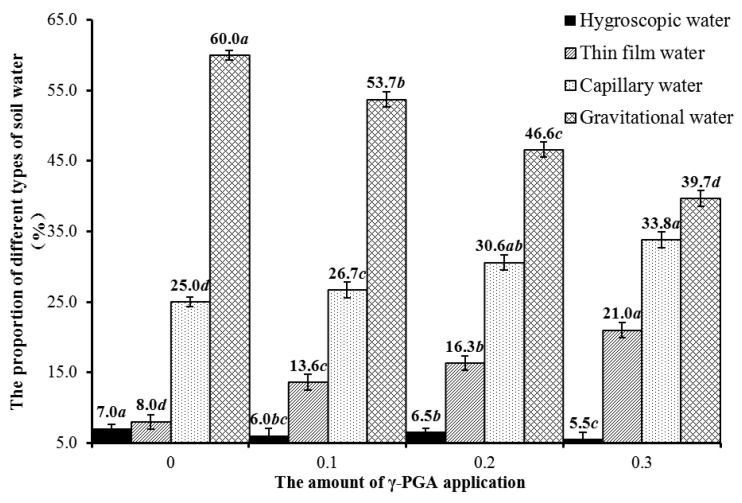
The proportion of different types of soil water. (The lowercase letters in Figure 8 represent the statistical results of ANOVA performed for the four main soil moistures under different γ-PGA content treatments; significant differences between treatments were considered when *p* < 0.05 level and non-significant when *p* ≥ 0.05.).

Figure 8 shows the analysis of the proportion of four main types of soil water in 1.35 g cm^−3^ γ-PGA-treated soil [40]. The γ-PGA application rates (0–0.3%) hardly affect the proportion of hygroscopic water. In contrast, the proportions of thin film water and capillary water increase with higher γ-PGA application rates. The increment of thin film water is higher than that of capillary water. Moreover, the proportion of gravitational water keeps a downward tendency as γ-PGA application increases. Overall, the proportion of unavailable soil water decreases when the application amount of γ-PGA increases by 3%.

In addition, the alphabetical order (ANOVA results) shown in Figure 8 reflects that the water change patterns of the four types under different γ-PGA content treatments showed significant differences (*p* < 0.05), in which the total amount of hygroscopic water and gravitational water decreased significantly with γ-PGA content, respectively, while the total amount of water of the other two types (thin film water and capillary water) increased significantly with the increase of γ-PGA content. The capillary water, as the main component of plant root water absorption, directly affects plant growth, which further indicates that the increase of γ-PGA content is beneficial to increase the proportion of its capillary water, so as to better absorb soil water and improve water utilization.

### 3.2. Effects of γ-PGA on Soil Infiltration Characteristics under Multi-Factors

#### 3.2.1. Cumulative Infiltration and Infiltration Rate

CK_1_ (1.3 g·cm^−3^) has the highest cumulative infiltration. In contrast, No. 7 (1.4 g·cm^−3^; 5–25 cm; 0.3%) has the lowest infiltration, which agrees with the study of mixed (0–10 cm) and layered (5 cm) applications [41]. This is because super absorbent polymer (SAP) in upper soil layer would restrict the downward movement of soil water. This restriction is more obvious than in mixed (10–20 cm) and layered (10 cm and 15 cm) applications and will increase with higher SAP concentration. No. 2 treatment and No. 3 treatment are less different with slight reduction of infiltration. No. 9, No. 8, and No. 4 treatments are less different, and their infiltration reductions are close to No. 7. To analyze the effects of multiple factors on cumulative infiltration, the least significant difference is performed among the factors and the levels (multivariate analysis of variance). It is clear that the bulk density and the application depth of γ-PGA have very significant influences (*p* < 0.01) on cumulative infiltration. Moreover, the γ-PGA application rate has significant influence (*p* < 0.05) (Table 6). According to LSD method, the influences of bulk density and the application depth of γ-PGA on infiltration are more significant than that of γ-PGA application rate. Therefore, bulk density and the application depth of γ-PGA are taken into consideration first for rational utilization of γ-PGA. The results are helpful for reducing agricultural cost and environmental protection.

An empirical model is set up through multiple regression analysis to describe the relationship among the cumulative infiltration, bulk density, the application depth of γ-PGA and γ-PGA application rates:

(15) $$I=\mu \cdot \rho^{a}\cdot h^{b}\cdot \gamma^{c}\cdot t^{x}$$

In Equation (15), I is the amount of cumulative infiltration; μ is the infiltration constant; ρ is bulk density, g·cm^−3^; h is the distance from the soil layer of soil mixed with γ-PGA to the surface soil, cm; γ is the content of γ-PGA (0.1, 0.2, and 0.3); t is infiltration time, min; and *a, b, c, x* are indicators.

Equation (16) is obtained through multiple regression analysis.

(16) $$I=0.71\cdot \rho^{-2.158}\cdot h^{0.025}\cdot \gamma^{-0.003}\cdot t^{0.619}$$

*RMSE* is 0.02 cm and *R*^2^ is 0.99 (*p* < 0.01). This illustrates that the empirical model has a good fit.

Equation (17) obtains the derivative of I and t in Equation (16).

(17) $$q=\frac{dI}{dt}=0.4395\cdot \rho^{-2.158}\cdot h^{0.025}\cdot \gamma^{-0.003}\cdot t^{-0.381}$$

In Equation (17), where q is infiltration rate, cm·min^−1^.

**Figure 9:**
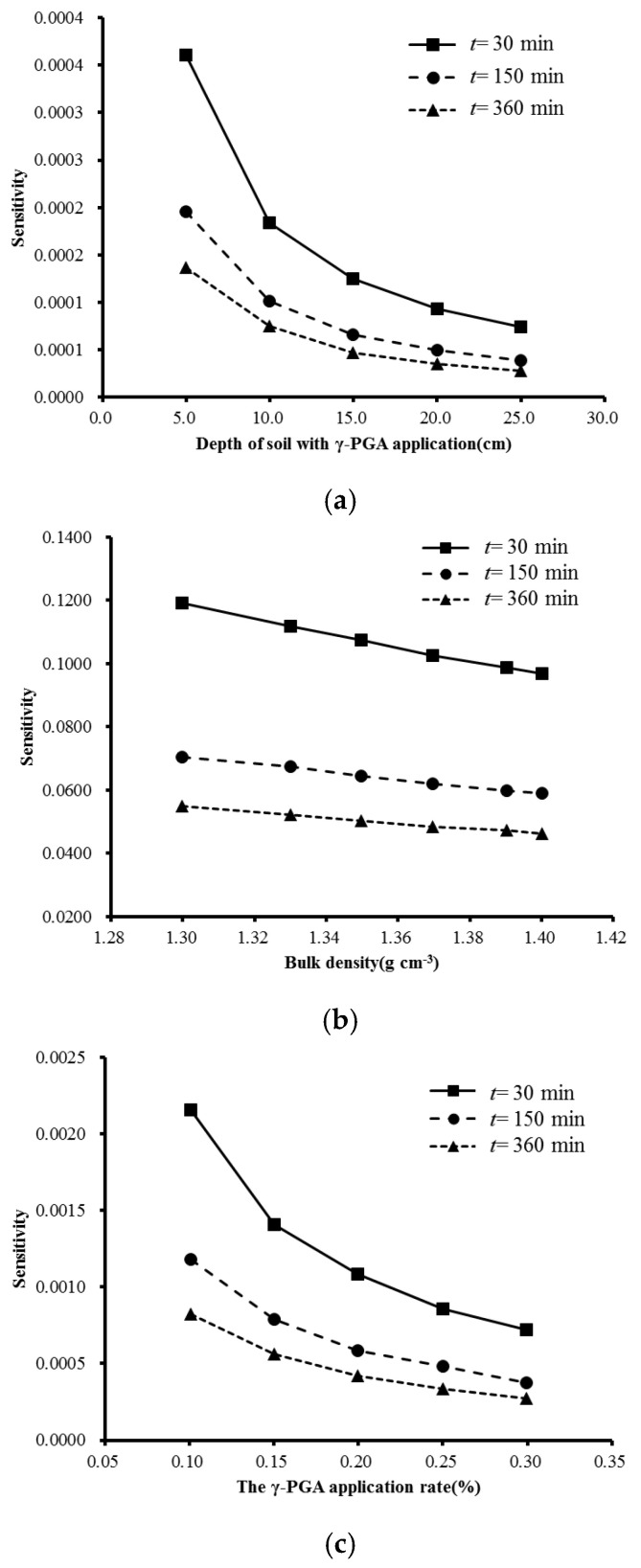
The sensitivity of various factors. (**a**) sensitivity vs. depth of soil with r-PGA application (cm). (**b**) sensitivity vs. bulk density (g cm^−3^). (**c**) sensitivity vs. the γ-PGA application rate (%).

The trend of infiltration rate affected by each factor is similar under each test treatment. Therefore, No.1 is used as an example to calculate the sensitivity of various factors (Figure 9a–c). There is a negative relationship between sensitivity and each factor. The variance of bulk density, the application depth of γ-PGA and application rate have a significant effect on the infiltration rate. Bulk density has the greatest effect on the infiltration rate and the application depth of γ-PGA exerts the smallest. Compared with the control group, the sensitivity of bulk density deceases by 0.03, 0.01, and 0.01 at 30 min, 150 min and 360 min of infiltration time; the sensitivity of the application depth of γ-PGA deceases by 0.0002, 0.0002, and 0.0001; and the sensitivity of γ-PGA application rate decreases by 0.001, 0.001, and 0.001, respectively.

#### 3.2.2. The Relationship between Wetting Front and Infiltration

The empirical model to describe the relationship among the wetting front distance, bulk density, the application depth of γ-PGA and γ-PGA application rate are as follows:

(18) $$H=\beta \cdot \rho^{e}\cdot h^{f}\cdot \gamma^{m}\cdot t^{y}$$

In Equation (18), where H is distance of the wetting front, cm; β is a constant; and *e, f, m, y* are indicators.

Equation (19) is obtained through multiple regression analysis.

(19) $$H=3.475\cdot \rho^{-2.607}\cdot h^{0.056}\cdot \gamma^{-0.025}\cdot t^{0.539}$$

*RMSE* and *R*^2^ are 0.03 cm and 0.98 (*p* < 0.01).

The Green-Ampt infiltration model is compared with the established empirical model. The comparison is based on the hypotheses of Green-Ampt infiltration model [42]. The relationship between the cumulative infiltration and wetting front distance are described in Equation (20).

(20) $$I=\left(\theta_{s}-\theta_{i}\right)H=\frac{H}{K_{1}}$$

where $\theta_{s}$ is saturation moisture content, cm^3^·cm^−3^; $\theta_{i}$ is initial moisture content, cm^3^·cm^−3^; $K_{1}$ is a constant obtained by fitting. As $\theta_{s}$ and $\theta_{i}$ are constants, there is a linear relationship between the cumulative infiltration and the distance of wetting front.

(21) $$\frac{H}{I}=4.89\cdot \rho^{-0.449}\cdot h^{0.031}\cdot \gamma^{-0.022}\cdot t^{-0.08}=K_{2}\cdot t^{-0.08}$$

According to Equation (21), the indicator of infiltration time (*t*) is close to zero. Therefore, there is a linear relationship between the cumulative infiltration and the distance of wetting front. The relative deviation of *K*_1_ and *K*_2_ ranges between 28% and 36%. This indicates that traditional Green-Ampt infiltration model is not suitable for layered soil with γ-PGA application [25].

#### 3.2.3. Model Validation

No. 10, No. 11, and No. 12 are used to verify the reliability of the empirical model. According to Table 7, the relative error between calculated value and test value of the cumulative infiltration and the distance of wetting front are lower than 10%. This shows that the established empirical model provides a good fit.

### 3.3. Effects of γ-PGA on Cl^−^Transport

**Figure 10:**
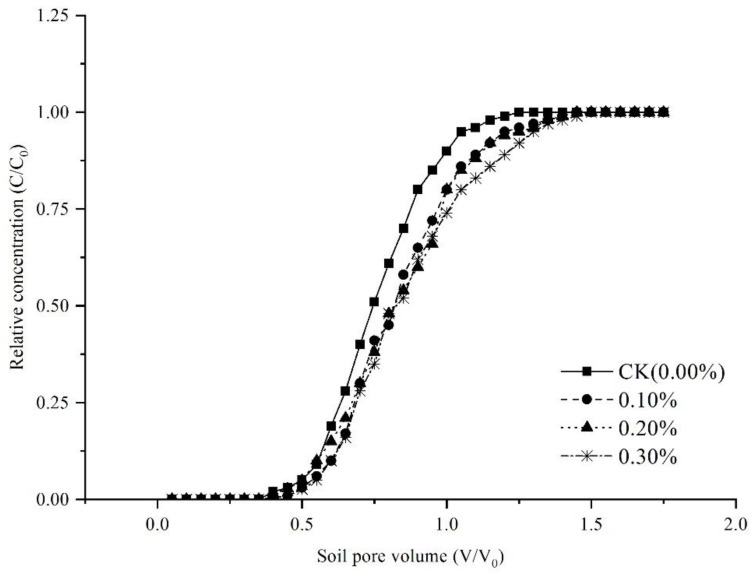
Soluble breakthrough curves (BTC).

When soil is treated with γ-PGA, solute breakthrough time becomes longer (Figure 10), presenting a smooth and S-shaped curve. As the γ-PGA application rate increases, the volume of initial penetration decreases, and the volume of complete penetration increases. STANMOD is used to inverse the solute transport parameters of convection-dispersion equation (CDE) (Table 8) [43]. The sum of squared residuals (SSQ) is close to zero. The initial penetration time t_0_ and the complete breakthrough time t_1_ of γ-PGA-treated soil are longer than that of CK and the hydrodynamic dispersion coefficient (D) and dispersion λ (λ = D/v) also increase. The reasons are as follows: First, the ability of γ-PGA to absorb water enhances the water-holding capacity of soil particles, promotes soil aggregation, and changes soil porosity [44]. Second, the hydrogels (from γ-PGA absorbing water) increase the viscosity of moisture and weaken the ability of capillary absorbing water and mechanical disperse. Thirdly, γ-PGA with a negatively charged carboxyl structure repels chloride ion in a free state.

## 4. Conclusions

Our laboratory experiments have simulated the effects of γ-PGA on soil-water infiltration, infiltration parameters, soil water-holding capacity, and soil solute (Cl^−^) transport. The results indicate that the infiltration capacity (*p* < 0.05) markedly decreased with the increase of γ-PGA application, and the stable infiltration rate (A) and infiltration sorption rate (S) decreased. γ-PGA application resulted in a higher percentage of available water.

The results of correlation analysis showed that γ-PGA content is positively correlated with *a* and *θs* and negatively correlated with steady infiltration rate A and n. The increase of γ-PGA content effectively increases the soil water-holding performance and improves the total water-holding capacity. It shows a significant inhibitory effect in the deep percolation characteristics of soil water, and the above effect is more significant for soil types with smaller soil capacity.

The LSD method is used to analyze multiple factors in cumulative infiltration. Considering the cost savings and ease of application in agriculture, the study recommends the use of γ-PGA treatment at 1.4 g cm^−3^ and 5–25 cm depth for soils with an application rate of 0.2%.

The results of solute transport parameters of the convection-dispersion equation (CDE) showed that, on the one hand, the penetration time interval of the γ-PGA-treated soil effectively increased, and the increase of its hydraulic dispersion coefficient (D) and dispersion degree (λ) effectively improved the water absorption capacity of the soil. On the other hand, due to the repulsion of chloride ions by the negatively charged carboxyl structure of γ-PGA in the free state, the upper surface of the soil quickly gathered higher water content. In comparison, the salt content in the soil is lower [45], effectively promoting the leaching of shallow soil salts. It can be widely applied in improving dry and saline agricultural lands.

## Figures and Tables

**Table 1:** Basic composition and physical properties characteristics of soils for the test.

The Soil Type	Volume Fraction/%			Median Particle Size	Initial Physical and Chemical Parameters of The Soil			
	Clay	Silt	Sand	D50	Initial Moisture Content	Saturated Water Content	Initial Salt Content	pH Value
	(<0.002 mm)	(≥0.002~0.02 mm)	(≥0.02~2 mm)	(μm)	(cm ^3^·cm^−3^)	(cm ^3^·cm^−3^)	(g·kg^−1^)	(-)
Sandy Loam	1.5	29.0	69.5	44.53	6.90	40.26	0.59 44.53 84.13	7.93

**Table 2:** Design of the orthogonal test.

Treatment	Bulk Density	Depth of γ-PGA Application	γ-PGA Content
	(g cm^−3^)	(cm)	(%)
No. 1	1.3	5–25	0.1
No. 2	1.3	15–35	0.2
No. 3	1.3	25–45	0.3
No. 4	1.35	5–25	0.2
No. 5	1.35	15–35	0.3
No. 6	1.35	25–45	0.1
No. 7	1.4	5–25	0.3
No. 8	1.4	15–35	0.2
No. 9	1.4	25–45	0.1
No. 10	1.32	8–28	0.12
No. 11	1.34	13–33	0.19
No. 12	1.38	18–38	0.28

**Table 3:** The soil hydraulic parameters of the soil with different γ-PGA application rates.

Treatments (γ-PGA Content%)	Parameters of VG Model (Inversed by HYDRUS-1D)					Measured	*R* ^2^	*RMSE*	*MAE*	*ME*
	Bulk Density	*θ_r_* (cm^3^·cm^3^)	*θ_s_* (cm^3^·cm^3^)	*α*	*n*	*K_s_* (cm·min^−1^)				
CK_1_ (0%)	1.30	0.031	0.40	0.0378	1.47	0.098	0.99	0.02	0.01	0.00
CK_2_ (0%)	1.35	0.031	0.39	0.0369	1.48	0.085	0.99	0.02	0.02	0.00
CK_3_ (0%)	1.40	0.031	0.38	0.0361	1.49	0.073	0.99	0.00	0.00	0.00
0.1%	1.30	0.032	0.42	0.0384	1.42	0.095	0.99	0.01	0.00	0.00
0.2%	1.30	0.032	0.43	0.0395	1.40	0.089	0.99	0.02	0.02	0.00
0.3%	1.30	0.033	0.43	0.0407	1.36	0.084	0.99	0.00	0.00	0.00
0.1%	1.35	0.032	0.40	0.0372	1.45	0.080	0.99	0.02	0.01	0.00
0.2%	1.35	0.032	0.42	0.0381	1.43	0.078	0.99	0.00	0.00	0.00
0.3%	1.35	0.032	0.42	0.039	1.4	0.074	0.99	0.00	0.00	0.00
0.1%	1.40	0.031	0.40	0.0366	1.47	0.071	0.99	0.00	0.02	0.00
0.2%	1.40	0.031	0.41	0.0370	1.43	0.069	0.99	0.00	0.00	0.00
0.3%	1.40	0.031	0.42	0.0376	1.41	0.067	0.99	0.00	0.00	0.00

Note: The sample data Size N = 297, the same as below. The experimental treatments in the control group CK relative to the γ-PGA application comparison is no γ-PGA(0%) treatment, and the treatments of 0.1% γ-PGA, 0.2% γ-PGA and 0.3% γ-PGA represent the application of γ-PGA as a percentage of the dry soil mass in the soil samples, respectively, in%.

**Table 4:** Parameters fitting and error analysis for Philip infiltration models.

Bulk Density (g·cm^−3^)	Parameters	CK (0%)	0.1%	0.2%	0.3%
1.30	*A*	0.03 ± 0.006	0.02 ± 0.002	0.02 ± 0.004	0.01 ± 0.003
	*S*	0.45 ± 0.001	0.45 ± 0.005	0.43 ± 0.001	0.41 ± 0.005
	*SSE*	0.43	0.35	0.31	0.24
	*RMSE*	0.22	0.19	0.18	0.15
	*R* ^2^	1.00	0.99	0.99	0.99
1.35	*A*	0.02 ± 0.002	0.02 ± 0.004	0.02 ± 0.001	0.01 ± 0.002
	*S*	0.41 ± 0.003	0.4 ± 0.008	0.4 ± 0.007	0.38 ± 0.003
	*SSE*	0.33	0.27	0.25	0.2
	*RMSE*	0.18	0.16	0.16	0.14
	*R* ^2^	0.99	0.99	0.99	0.99
1.40	*A*	0.02 ± 0.001	0.02 ± 0.002	0.01 ± 0.004	0.01 ± 0.007
	*S*	0.38 ± 0.003	0.37 ± 0.006	0.37 ± 0.005	0.36 ± 0.003
	*SSE*	0.25	0.22	0.2	0.17
	*RMSE*	0.16	0.15	0.14	0.13
	*R* ^2^	0.99	0.99	0.99	0.99

**Table 5:** The relationship between the γ-PGA application rate and Philip model parameters.

Bulk Density (g·cm^−3^)	Parameters	Fitting Formula	*R* ^2^	*SSE*	*RMSE*
1.30	*A*	$A=0.03e^{-2.28x}$	0.99	0.038	0.021
	*S*	$S=0.45e^{-0.30x}$	0.88	0.108	0.064
1.35	*A*	$A=0.02e^{-2.28x}$	0.97	0.052	0.033
	*S*	$S=0.41e^{-0.25x}$	0.86	0.103	0.072
1.40	*A*	$A=0.02e^{-2.18x}$	0.98	0.051	0.029
	*S*	$S=0.38e^{-0.21x}$	0.96	0.060	0.027

Note: ‘*x*’ represents the different amount of γ-PGA application in the soil column.

**Table 6:** Influence of different factors in different levels of the cumulative infiltration.

Level	Bulk Density (g cm^−3^)	The Depth of γ-PGA Application (cm)	The Amount of γ-PGA Application (%)
1	18.2 ± 0.760 Aa	15.2 ± 0.634 Aa	16.2 ± 1.133 Aa
2	16 ± 0.668 Bb	16.4 ± 0.689 Bb	16.0 *±* 0.982 Ba
3	13.7 ± 0.572 Cc	16.5 ± 0.844 Cc	15.9 ± 1.037 Ba

Note: Different small letters mean significant difference (*p* < 0.05) and different capital letters mean extremely significant difference (*p* < 0.01).

**Table 7:** Empirical model validation.

Treatment	No. 10					
Time (min)	Cumulative Infiltration of Formula (16)			Wetting Front Distance of Formula (19)		
	Experimental Data (cm)	Calculated Value (cm)	Relative Deviation (%)	Experimental Data (cm)	Calculated Value (cm)	Relative Deviation (%)
15	2.4	2.1	−13.3	8.9	8.6	−3.4
20	2.7	2.5	−10.4	9.9	10.0	1.4
30	3.4	3.1	−7.0	12.1	12.5	3.3
45	4.2	4.0	−4.3	15.2	15.5	2.2
60	5.0	4.8	−2.9	17.0	18.1	6.7
90	6.4	6.2	−2.2	20.7	22.6	9.1
120	7.6	7.4	−2.9	25.0	26.4	5.4
150	8.9	8.5	−4.2	28.3	29.7	5.1
180	10.1	9.5	−5.8	32.0	32.8	2.5
240	12.6	11.4	−9.3	40.0	38.3	−4.3
300	14.1	13.1	−7.2	47.0	43.2	−8.1
360	16.1	14.6	−9.2	52.8	47.7	−9.8
Treatment	No. 11					
Time (min)	Cumulative Infiltration of Formula (16)			Wetting Front Distance of Formula (19)		
	Experimental Data (cm)	Calculated Value (cm)	Relative Deviation (%)	Experimental Data (cm)	Calculated Value (cm)	Relative Deviation (%)
15	2.3	2.0	−14.1	8.7	8.39	−3.5
20	2.6	2.3	−11.1	9.6	9.80	2.1
30	3.2	3.0	−7.5	11.8	12.20	3.4
45	4.0	3.8	−5.0	14.3	15.18	6.1
60	4.8	4.6	−4.0	16.6	17.72	6.8
90	6.1	5.9	−3.8	20.1	22.05	9.7
120	7.4	7.1	−4.6	24.1	25.75	6.8
150	8.6	8.1	−6.0	27.7	29.04	4.8
180	9.8	9.1	−7.5	31.6	32.04	1.4
240	11.5	10.8	−6.0	39.6	37.41	−5.5
300	12.9	12.4	−3.4	45	42.19	−6.2
360	13.8	13.9	1.2	50.6	46.6	−8.0
Treatment	No. 12					
Time/min	Cumulative Infiltration of Formula (16)			Wetting Front Distance of Formula (19)		
	Experimental Data (cm)	Calculated Value (cm)	Relative Deviation (%)	Experimental Data (cm)	Calculated Value (cm)	Relative Deviation (%)
15	2.1	1.8	−12.5	8.2	7.84	−4.4
20	2.4	2.2	−9.3	9.4	9.2	−2.6
30	3.0	2.8	−5.5	11.5	11.4	−1.3
45	3.7	3.6	−2.8	13.9	14.2	2.0
60	4.4	4.3	−1.5	15.8	16.6	4.8
90	5.6	5.5	−1.2	19.6	20.6	5.1
120	6.7	6.6	−2.0	23.2	24.1	3.7
150	7.8	7.6	−3.3	26.4	27.1	2.8
180	8.9	8.5	−4.8	29.8	29.9	0.4
240	10.9	10.1	−7.4	35.2	35.0	−0.7
300	12.4	11.6	−6.4	41.6	39.4	−5.3
360	13.0	13.0	0.4	48.2	43.5	−9.8

**Table 8:** The solute transport fitting parameters by CDE.

γ-PGA (%)	*t*_0_	*t*_1_	*R*	*λ* cm^−1^	*v*	*D*	*R* ^2^
	min	min			cm·min^−1^	cm^2^·min^−1^	
CK (0)	304.3	507.1	0.74	0.45	0.03	0.02	0.99
0.1	358.0	716.0	0.81	0.35	0.03	0.01	0.99
0.2	494.4	1236.0	0.86	0.62	0.03	0.02	0.99
0.3	562.8	1407.0	0.80	0.77	0.03	0.02	0.99

Note: *t*_0_ represents the initial penetration time; *t*_1_ represents the complete breakthrough time; *R* represents the blocking factor; *λ* represents the dispersion, which represents equal to *D*/*v*; *v* represents the average pore water flow rate; D represents the dispersion coefficient.

## Data Availability

The original contributions presented in the study are included in the article, further inquiries can be directed to the corresponding author.

## References

[1] Heng T., Feng G., Yang L.L., He X.L., Yang G., Li F.D., Xu X., Feng Y. (2021). Soil salt balance in a cotton field under drip irrigation and subsurface pipe drainage systems. Agron. J..

[2] Minhas P.S., Ramos T.B., Ben-Gal A., Pereira L.S. (2020). Coping with salinity in irrigated agriculture: Crop evapotranspiration and water management issues. Agric. Water Manag..

[3] Xu H., Song J. (2022). Drivers of the irrigation water rebound effect: A case study of Hetao irrigation district in Yellow River basin, China. Agric. Water Manag..

[4] He Z., Gong K., Zhang Z., Dong W., Feng H., Yu Q., He J. (2022). What is the past, present, and future of scientific research on the Yellow River Basin?—A bibliometric analysis. Agric. Water Manag..

[5] Lan H., Peng J., Zhu Y., Li L., Pan B., Huang Q., Li J., Zhang Q. (2022). Research on geological and surfacial processes and major disaster effects in the Yellow River Basin. Sci. China Earth Sci..

[6] Zhao C., Zhang L., Zhang Q., Wang J., Wang S., Zhang M., Liu Z. (2022). The effects of bio-based superabsorbent polymers on the water/nutrient retention characteristics and agricultural productivity of a saline soil from the Yellow River Basin, China. Agric. Water Manag..

[7] Lin Q., Wang S., Li Y., Riaz L., Yu F., Yang Q., Han S., Ma J. (2022). Effects and mechanisms of land-types conversion on greenhouse gas emissions in the Yellow River floodplain wetland. Sci. Total Environ..

[8] Lai J., Liu T., Luo Y. (2022). Evapotranspiration partitioning for winter wheat with shallow groundwater in the lower reach of the Yellow River Basin. Agric. Water Manag..

[9] Zhu H., Yang J.S., Yao R.J., Gao S., Cao Y.F., Sun Y.P. (2019). Effects of partial substitution of organic nitrogen for inorganic nitrogen in fertilization on salinity and nitrogen utilization in salinized coastal soil. Chin. J. Eco-Agric..

[10] Li Y., Wang W.Y., Wang Q.J. (2001). A breakthrough thought for water saving and salinity control in arid and semi-arid area under film trickle irrigation. Irrig. Drain..

[11] Guo J., Shi W., Wang P., Hao Q., Li J. (2022). Performance characterization of γ-poly (glutamic acid) super absorbent polymer and its effect on soil water availability. Arch. Agron. Soil Sci..

[12] Zhang L., Wei Z., Wang L., Sun Y., Pei J., Wang J., Gao J., Zhang L., Shi Y. (2022). Fate of urea and ammonium sulfate in the plant and soil system as affected by poly-γ-glutamic acid. J. Soil Sci. Plant Nutr..

[13] Xu Z., Lei P., Feng X., Xu X., Xu H., Yang H., Tang W. (2013). Effect of poly(γ-glutamic acid) on microbial community and nitrogen pools of soil. Acta Agric. Scand. Sect. B Soil Plant Sci..

[14] Xu Z., Wan C., Xu X., Feng X., Xu H. (2013). Effect of poly (γ-glutamic acid) on wheat productivity, nitrogen use efciency and soil microbes. J. Soil Sci. Plant. Nutr..

[15] Zhang L., Yang X., Gao D., Wang L., Li J., Wei Z., Shi Y. (2017). Efects of poly-γ-glutamic acid (γ-PGA) on plant growth and its distribution in a controlled plant-soil system. Sci. Rep..

[16] Wang X., Sun R., Tian Y., Guo K., Sun H., Liu X., Chu H., Liu B. (2020). Long term phytoremediation of coastal saline soil reveals plant species-specific patterns of microbial community recruitmnent. mSystems.

[17] Yang Z.H., Dong C.D., Chen C.W., Sheu Y.T., Kao C.M. (2018). Using poly-glutamic acid as soil-washing agent to remediate heavy metal-contaminated soils. Environ. Sci. Pollut. Res..

[18] Peng Y.P., Chang Y.C., Chen K.F., Wang C.H. (2020). A field pilot-scale study on heavy metal contaminated soil washing by using an environmentally friendly agent—poly-r-glutamic acid (r PGA). Environ. Sci. Pollut. Res..

[19] Pang X., Lei P., Feng X., Xu Z., Xu H., Liu K. (2018). Poly-γ-glutamic acid, a bio-chelator, alleviates the toxicity of Cd and Pb in the soil and promotes the establishment of healthy *Cucumis sativus* L. seedling. Environ. Sci. Pollut. Res..

[20] Liang J., Shi W., He Z., Pang L., Zhang Y. (2019). Effects of poly-γ-glutamic acid on water use efficiency, cotton yield, and fiber quality in the sandy soil of souther Xinjiang, China. Agric. Water Manag..

[21] Tang D., Mao L., Zhi Y.E., Zhang J.Z., Zhou P., Chai X.T. (2014). Investigation and canonical correspondence analysis of salinity contents in secondary salinization greenhouse soils in Shanghai suburb. Environ. Sci..

[22] Wu Q., Cui Y., Li Q., Sun J. (2015). Effective removal of heavy metals from industrial sludge with the aid of a biodegradable chelating ligand GLDA. J. Hazard Mater..

[23] Giri B., Kapoor R., Mukerji K.G., Mukerji K.G., Manorachari C., Singh J. (2002). VA mycorrhizal techniques/VAM Technology in establishment of plants under salinity stress conditions. Techniques in Mycorrhizal Studies.

[24] Song Y., Yang X., Yang S., Wang J. (2019). Transcriptome sequencing and functional analysis of *Sedum lineare* Thunb. upon salt stress. Mol. Genet. Genom..

[25] Sharda A.K. (1977). Influence of soil bulk density on horizontal water infiltration. Soil Res..

[26] Wang H.T. (2008). Dynamics of Fluid Flow and Contaminant Transport in Porous Media.

[27] Islam M.N., Bell R.W., Barrett-Lennard E.G., Maniruzzaman M. (2022). Shallow surface and subsurface drains alleviate waterlogging and salinity in a clay-textured soil and improve the yield of sunflower in the Ganges Delta. Agron. Sustain. Dev..

[28] Hu Y., Kang S., Ding R., Zhao Q. (2021). A crude protein and fiber model of alfalfa incorporating growth age under water and salt stress. Agric. Water Manag..

[29] Zeng J., Fei L., Chen L., Yang Y. (2018). Effects of γ-PGA on soil structure and water-holding characteristics. J. Soil Water Conserv..

[30] Mualem Y. (1976). A new model for predicting the hydraulic conductivity of unsaturated porous media. Water Resour. Res..

[31] Van Genuchten M.T. (1980). A closed-form equation for predicting the hydraulic conductivity of unsaturated soils. Soil Sci. Soc. Am. J..

[32] Van Genuchten M.T., Leij F.J., Yates S.R. (1991). The RETC Code for Quantifying the Hydraulic Functions of Unsaturated Soils.

[33] Karamov R., Akhatov I., Sergeichev I.V. (2022). Prediction of Fracture Toughness of Pultruded Composites Based on Supervised Machine Learning. Polymers.

[34] Yichen Z.H.U., Guangming L.I.U., Xin L.I.U., Feng P.E.I., Xu T.I.A.N., Chao S.H.I. (2019). Investigation on Interrelation of Field Corrosion Test and Accelerated Corrosion Test of Grounding Materials in Red Soil Environment. J. Chin. Soc. Corros. Prot..

[35] Philip J.R. (1957). The theory of infiltration: 4. Sorptivity and algebraic infiltration equations. Soil Sci..

[36] Simunek J., Van Genuchten M.T., Sejna M. (2005). The HYDRUS-1D Software Package for Simulating the One-Dimensional Movement of Water, Heat, and Multiple Solutes in Variablysaturated Media.

[37] Guo J., Shi W., Wen L., Shi X., Li J. (2020). Effects of a super-absorbent polymer derived from poly-γ-glutamic acid on water infiltration, field water capacity, soil evaporation, and soil water-stable aggregates. Arch. Agron. Soil Sci..

[38] Liang J.P., Shi W.J. (2021). Poly-γ-glutamic acid improves water-stable aggregates, nitrogen and phosphorus uptake efficiency, water-fertilizer productivity, and economic benefit in barren desertified soils of Northwest China. Agric. Water Manag..

[39] Shi X.X., Shi W.J., Pang L.N., Wen L.J., Gao Z.Y. (2020). Effects of Y-Polyglutamic Acid on Soil Water and Nitrogen Transport Characteristics. J. Soil Water Conserv..

[40] Huang Q.Y., Tang S.H., Li P., Fu H.T., Zhang M., Huang X., Yi Q., Zhang F.B. (2016). Agronomic effects of coating material γ-polyglutamic acid on Chinese flowering cabbage. J. Plant Nutr. Fertil..

[41] Bai W., Song J., Li M., Wang Y., Wu Y., Liu B., Wang C., Wang X. (2009). Effect of super absorbent polymer on vertical infiltration characteristics of soil water. Trans. Chin. Soc. Agric. Eng..

[42] Green W.H., Ampt G.A. (1911). Studies on soil physics. J. Agric. Sci..

[43] Van Genuchten M.T., Šimunek J., Leij F.J., Toride N., Šejna M. (2012). STANMOD: Model use, calibration, and validation. Trans. ASABE.

[44] Alkhasha A., Al-Omran A., Aly A. (2018). Effects of biochar and synthetic polymer on the hydro-physical properties of sandy soils. Sustainability.

[45] Han Y.G., Wu H.F., Yang P.L., Ding T., Xin X.H., Sun M., Song H., Li Y.Y. (2013). Dynamic effects of super absorbent polymer on physical properties and water infiltration of soil. Agric. Res. Arid. Areas.

